# Case report of amniotic fluid embolism coagulopathy following abortion; use of viscoelastic point-of-care analysis

**DOI:** 10.1186/s12884-019-2680-1

**Published:** 2020-01-03

**Authors:** Halley P. Crissman, Charisse Loder, Carlo Pancaro, Jason Bell

**Affiliations:** 1 Department of Obstetrics and Gynecology, University of Michigan, 1500 E. Medical Center Dr., Ann Arbor, MI 48109 USA; 2 University of Michigan Program on Women’s Healthcare Effectiveness Research (PWHER), 1500 E. Medical Center Dr., Ann Arbor, MI 48109 USA; 3 Department of Anesthesiology, University of Michigan, 1500 E. Medical Center Dr., Ann Arbor, MI 48109 USA

**Keywords:** Abortion, Amniotic fluid embolism, Viscoelastic point-of-care analysis, Thromboelastography, Coagulopathy, Case report

## Abstract

**Background:**

Amniotic fluid embolism (AFE) is a rare, life threatening obstetric complication, often associated with severe coagulopathy. Induced abortions are extremely safe procedures however complications including AFE can occur.

**Case presentation:**

A 29-year-old previously healthy woman, gravida 1 para 0, presented for a scheduled second trimester induced abortion via dilation and evacuation at 22-weeks gestation. The case was complicated by a suspected AFE with associated profound coagulopathy. Viscoelastic point-of-care coagulation analysis was used to successfully and swiftly guide management of her coagulopathy.

**Conclusion:**

AFE can occur in the setting of induced abortion. This case report suggests viscoelastic point-of-care coagulation analyzers may aid in the management of pregnancy-related coagulopathy by providing faster coagulation assessment than laboratory testing, and facilitating timely, targeted management of coagulopathy.

## Background

Amniotic fluid embolism (AFE) is a rare complication of pregnancy associated with significant morbidity and mortality. AFE involves a complex sequence of abnormal activation of proinflammatory mediators in the setting of disruption of the maternal-fetal interface, typically presenting as sudden cardiorespiratory collapse, followed in the majority of cases by disseminated intravascular coagulopathy [1].

The incidence of AFE in the setting of legal induced abortion is unknown but is likely low given the very low morbidity and mortality associated with legal induced abortion [2]. Analysis of the 2011–2013 Pregnancy Mortality Surveillance System attributed 5.5% of maternal deaths in the United States to AFE, with 1 of the 111 reported AFE related maternal deaths occurring following an abortion [3].

We present the case of a second trimester surgical abortion complicated by a suspected AFE, where profound coagulopathy was successfully managed using targeted blood component repletion guided by viscoelastic point-of-care coagulation analysis. Thromboelastography (TEG®; Haemonetics Corp, Braintree, MA) and rotational thromboelastography (ROTEM®; Tem International GmbH; Munich; Germany) are real-time, point of care techniques for assessing specific viscoelastic properties of whole blood as it clots under low shear conditions [4]. In comparison to traditional laboratory coagulation tests, viscoelastic point-of-care coagulation analyzers provide more rapid results and facilitate targeted blood component therapy [4, 5]. While viscoelastic point-of-care coagulation analyzers have been used for years in trauma, cardiac, and liver surgery, their use in management of pregnancy-related coagulopathy is not yet widespread [4].

We present this case to highlight the potential role for ROTEM® viscoelastic point-of-care coagulation analysis in directed and timely management of AFE associated coagulopathy, including as a rare complication of legal induced second trimester surgical abortions.

## Case presentation

The patient is a 29-year-old previously healthy female, gravida 1 para 0, who presented for a scheduled induced abortion via dilation and evacuation at 22-weeks gestation, in the setting of a pregnancy complicated by a fetus with trisomy 22 confirmed on amniocentesis and multiple fetal anomalies. Workup prior to the procedure included a complete blood count which revealed a hemoglobin of 12.1 g/deciliter and platelets of 134 ×  10^3^/μl. No history of pre-pregnancy thrombocytopenia was noted. The patient’s pre-procedure systolic blood pressures ranged from 80 to 100 s mmHg.

The day prior to the procedure the patient had osmotic cervical dilators placed in the office. On the day of the procedure a singleton fetus with fetal heart tones was confirmed by ultrasound, monitored anesthesia care was administered with fentanyl, midazolam and propofol in an operating room, osmotic cervical dilators were removed, and a paracervical block with 1% lidocaine was performed. The patient then underwent ultrasound-guided dilation and evacuation using electric suction curettage and Bierer forceps. All fetal parts and the placenta were accounted for at the end of the case, with an estimated blood loss of 100 ml and no apparent complications. No hypoxia, tachycardia, or hypotension were noted in the operating room at the time of the procedure.

In the post-anesthesia care unit 15 min post-operatively, the patient experienced moderate vaginal bleeding (visually estimated to be 200 ml) with normal vital signs. The leading diagnosis for additional blood loss at this time was uterine atony; methylergonovine 200 micrograms intra-muscular was administered and a 500 ml crystalloid bolus was given. Forty-five minutes after the procedure moderate vaginal bleeding (visually estimated to be additional 200 ml) was noted again. On assessment vital signs were normal, transabdominal ultrasound revealed a thin endometrial stripe and no fluid posterior to the uterus to suggest intra-abdominal hemorrhage. Firm uterine tone was noted on bimanual exam. Rectal misoprostol (800 micrograms) was administered.

Finally, 75 min post-operatively, the patient became acutely hypotensive (54/42 mmHg). Anesthesia and gynecology teams were immediately called to the bedside. An additional crystalloid fluid bolus was initiated and the decision was made to transfer the patient back to the operating room for evaluation, considering a differential diagnosis for bleeding including retained products of conception, uterine atony, cervical laceration, or coagulopathy.

In the operating room, a complete blood count, activated partial thromboplastin time (aPTT), and International Normalized Ratio (INR) were obtained. Monitored anesthesia care was again administered. A 1 × 2 cm piece of clot was removed from the endometrial canal on bimanual exam. Firm uterine tone was noted. Gritty texture and no ongoing bleeding was noted with repeated curettage with a suction cannula. No cervical lacerations were noted, however, hemostatic agents were applied to two site of oozing on the external *os*. Blood loss was estimated to be 750 ml total at this time.

The patient was transferred back to the post-anesthesia care unit, where an additional dose of intramusclular methylergonovine 200 micrograms was administered given continued moderate bleeding while awaiting lab results. Intra-operative lab results returned showing mild anemia, worsened thrombocytopenia, and abnormal elevation of INR and aPTT (Table 1, Lab 1). Clinical suspicion at this time was for possible AFE, given an episode of significant hypotension and coagulopathy out of proportion to blood loss. STAT repeat labs including a fibrinogen level were ordered, in addition to a ROTEM®.

**Table 1:** Timeline of laboratory results, ROTEM® results, and interventions in order of time when intervention or testing was initiated

Event or Intervention	Time Obtained	Processing Time^a^ (minutes)	CBC^b^	aPTT (seconds)	INR	Fibrinogen (milligrams/deciliter)/FIBTEM A10 (mm)	EXTEM Clotting Time (CT) and A10
Pre-Op			12.1 134				
Event	Dilation and evacuation completed at 08:34.						
Event	Ongoing vaginal bleeding, episode of hypotension, return to the OR at 10:28.						
Lab 1	10:51	88	10.4 84	38.0	1.7		
Lab 2	13:08	132	10.6 93	38.5	2.0	Fibrinogen < 25	
ROTEM® 1	13:30					Undetectable FIBTEM value	CT = 359 s, A10 = 16 mm
Intervention	At time of ROTEM® 1 result, initiated 3 g concentrated fibrinogen, 1 unit FFP, 1 × 5-pack platelets.						
Lab 3	14:21	72	9.5 53	34.4	1.4	Fibrinogen 49	
ROTEM® 2	14:21					2 mm FIBTEM (low amplitude)	Improvement in EXTEM. CT = 187 s, A10 = 20 mm
Intervention	At time of ROTEM® 2 result, initiated 1 g concentrated fibrinogen, 2 units of cryoprecipitate, 3 units of FFP, 1 × 5-pack platelets, 2 g TXA.						
Lab 4	15:47	57	6.2 81	22.4	1.0	Fibrinogen 255	
Intervention	1 unit cryoprecipitate, 2 units red blood cells given between obtaining Lab 4 and receiving the results.						
ROTEM® 3	16:44					13 mm FIBTEM (normal amplitude)	Normal EXTEM. CT = 60 s, A10 = 45 mm
Lab 5	17:01	53	8.7 79	26.6	1.0	Fibrinogen 243	
Intervention	2 × 5-pack platelets.						
	22:21	34	8.8 141	25.3	1.0	Fibrinogen 283	

^a^Processing time (minutes) reflects the time from lab collection to the full panel of labs resulting in the electronic medical record system

^b^ Hgb Platelets

Complete blood count (CBC) with hemoglobin (Hgb) measured in grams/deciliter, platelets in unit × 10^3^/μl. Activated partial thromboplastin time (aPTT), International Normalized Ratio (INR), clot amplitude at 10 min (A10), clotting time (CT), rotational thromboelastography (ROTEM®), fresh frozen plasma (FFP), tranexamic acid (TXA)

**Fig. 1:**
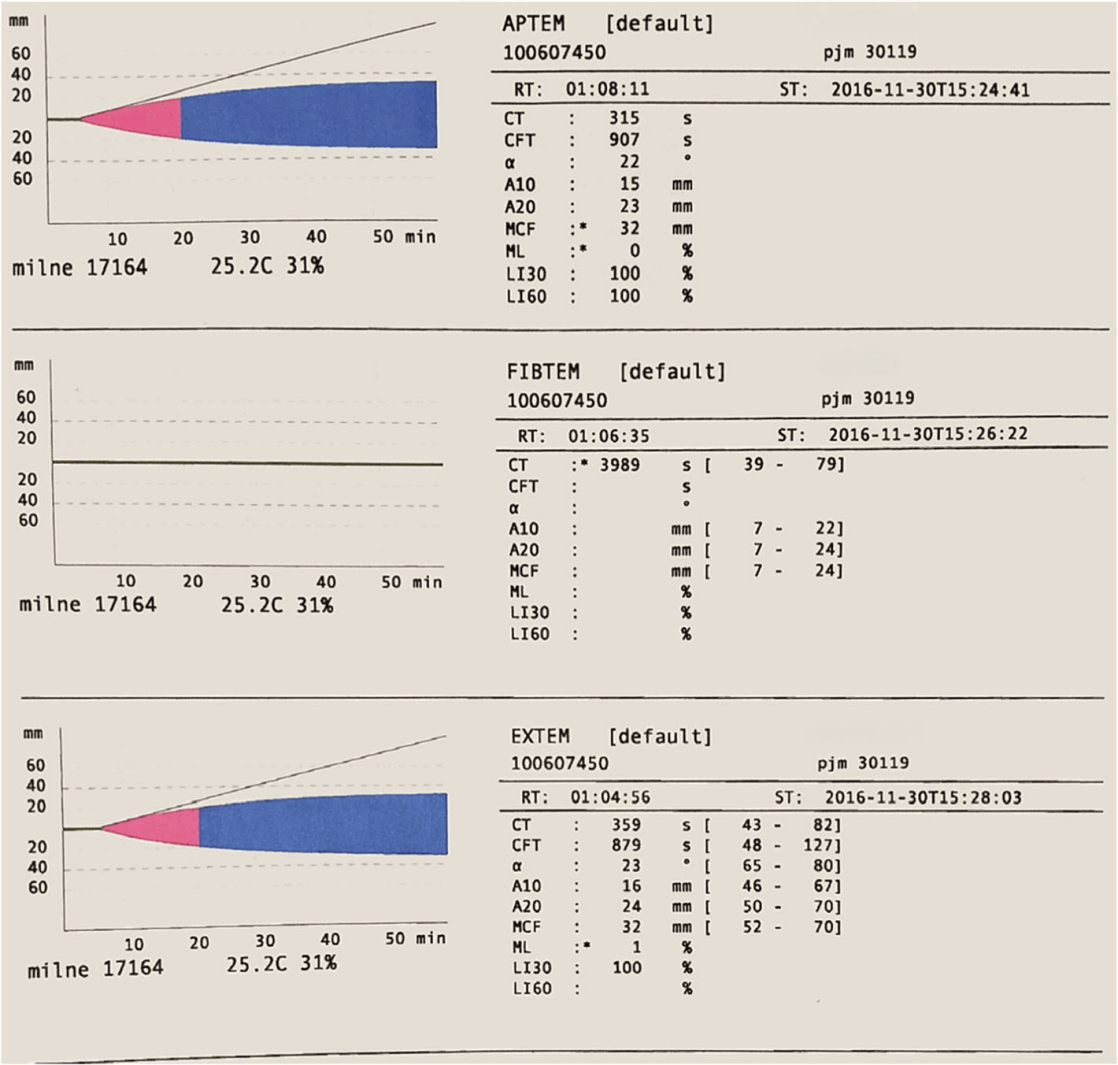
ROTEM® 1: ROTEM® results show very low amplitude fibrinogen; FIBTEM A10 is undetectable. EXTEM results show prolonged CT (359 s) and low clot A10 in the EXTEM (16 mm)

**Fig. 2:**
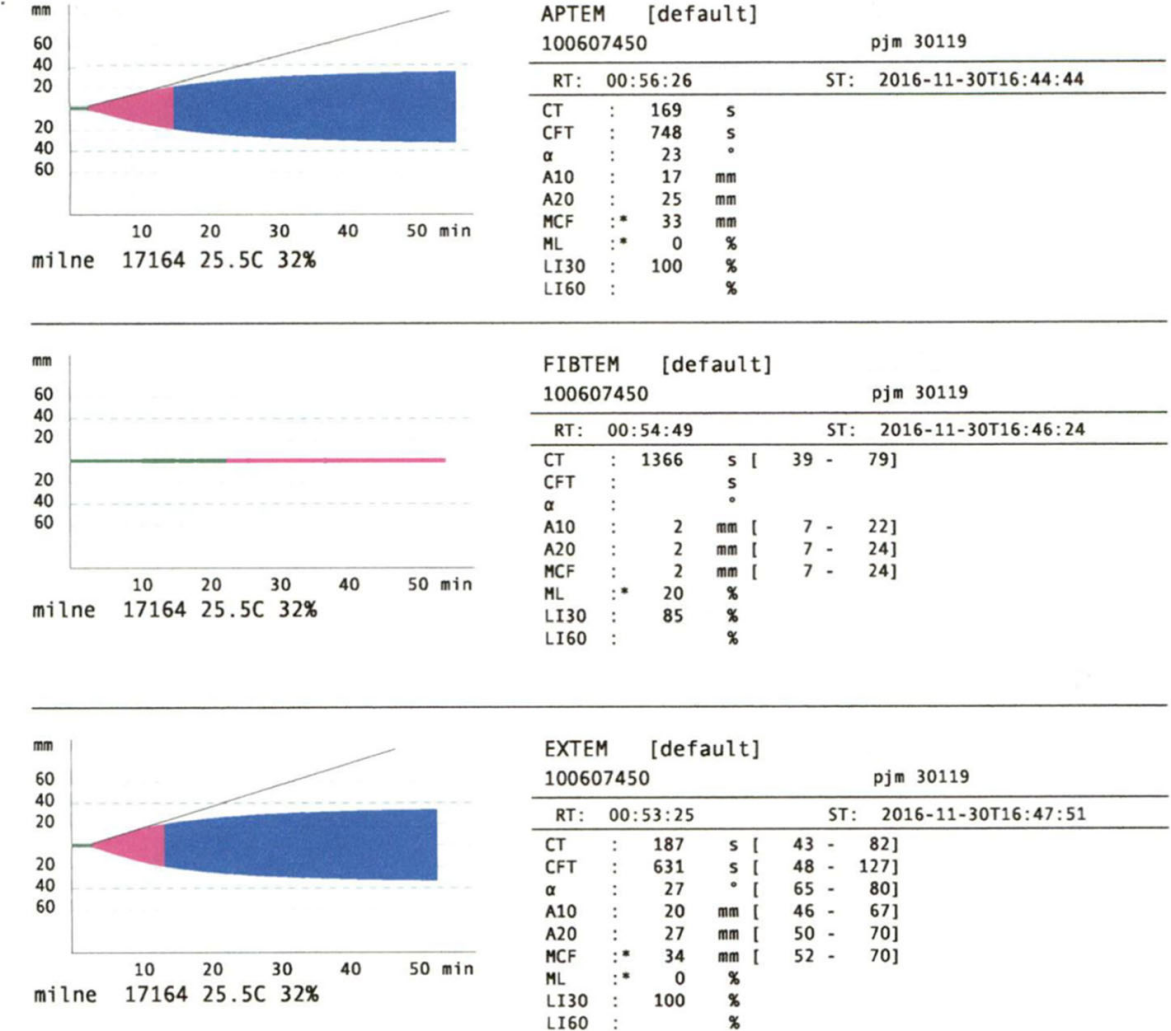
ROTEM® 2: ROTEM® results continue to show severe hypofibrinogemia; FIBTEM A10 value of 2 mm. EXTEM results suggest some improvement in CT (187 s) and A10 (20 mm)

ROTEM® results (Fig. 1) showed very low amplitude fibrinogen, reflected in the low amplitude at 10 min (A10) in the FIBTEM channel, and prolonged clotting time (CT) (359 s) and reduced clot amplitude A10 in the EXTEM (16 mm) channel. Concentrated fibrinogen and transfusion of fresh frozen plasma and platelets were initiated immediately. Additional blood products were ordered from the blood bank. Fifty minutes after the initial ROTEM®, a repeat ROTEM® and repeat STAT labs were sent; at this time the full results were not yet available from the previous labs sent for analysis. More than 2 h after the first infusion of concentrated fibrinogen was initiated based on the first ROTEM® results, the first STAT fibrinogen level resulted as less than 25 mg/deciliter (Table 1, Lab 2). By the time these labs resulted, a second ROTEM® had been run and continued to show severe hypofibrinogemia, as shown in the FIBTEM A10 value (2 mm), and improvement in EXTEM CT (187 s) (Fig. 2).

**Fig. 3:**
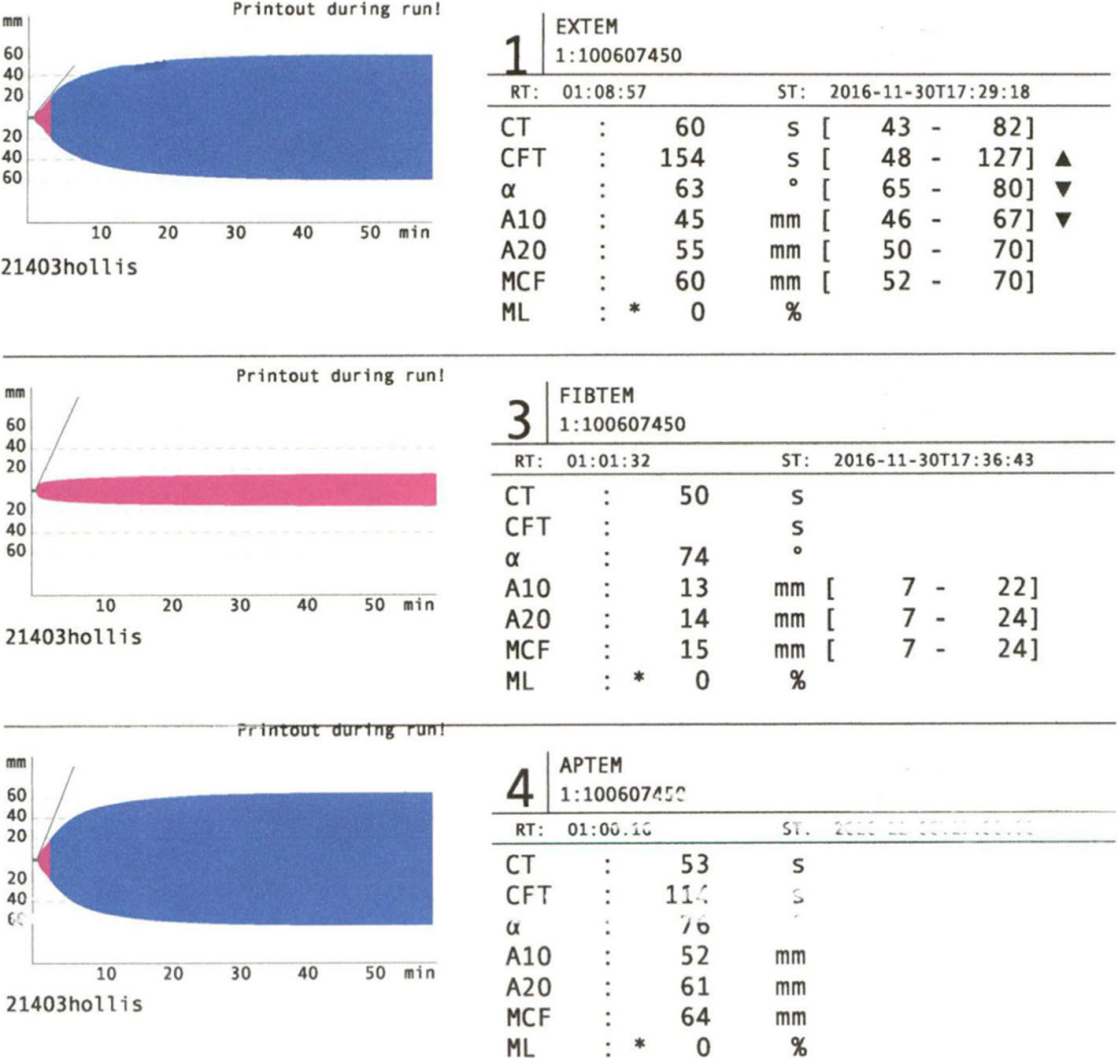
ROTEM® 3: ROTEM® show normalization of fibrinogen levels; FIBTEM A10 of 13 mm. EXTEM results show normalization of CT (60 s), and A10 (45 mm)

Concurrently, the patient developed hypoxia, was placed on a non-rebreather mask and the surgical intensivist team was consulted. Labs sent at the time of the second ROTEM® confirmed ongoing coagulopathy (Table 1, Lab 3). After receiving 4 g concentrated fibrinogen, 2 units of cryoprecipitate, 4 units of fresh frozen plasma, 2 × 5-pack platelets, and 2 g of tranexamic acid, repeat labs were obtained; one unit cryoprecipitate and 2 units of packed red blood cells were given between obtaining the labs and receiving the results. Upon arrival to the surgical intensive care unit, labs showed worsened anemia (not yet reflecting the 2 units of packed red blood cells given after the labs were obtained), improving thrombocytopenia, normalization of aPTT and INR, and normalization of fibrinogen (Table 1, Lab 4). Repeat ROTEM® showed normalization of fibrinogen levels (FIBTEM A10 = 13 mm), CT (60 s), and EXTEM A10 (45 mm) (Fig. 3).

After transfer to the surgical intensive care unit, a chest radiograph showed diffuse hazy opacification predominant in the peri-hilar regions and lower lungs. The patient received heated high flow nasal cannula secondary to desaturations and 2 × 5-pack platelets for thrombocytopenia. She remained hemodynamically stable, her hypoxia resolved, and she was discharged on room air on post-operative day two. She was doing well and asymptomatic at her two-week post-operative appointment.

## Discussion

This case demonstrates the potential advantages of the use of viscoelastic point-of-care coagulation analyzers for the management of coagulopathy in the setting of a suspected AFE. While legal induced abortions are safe procedures which can be performed in an outpatient setting in most cases, our case also serves as a reminder to clinicians that rare pregnancy-related complications including AFE can occur [2].

When confronted with pregnancy-related cardiovascular collapse and profound coagulopathy, AFE should be considered in the differential diagnosis, along with pulmonary embolism, myocardial infarction, anesthetic complication, anaphylaxis, and acute massive blood loss [6]. When coagulopathy is associated with cardiovascular dysfunction, and massive blood loss has been ruled out, as in our case, AFE should be suspected. Classically a clinical triad of hypoxia, hypotension, and coagulopathy is seen in AFEs, with common symptoms including dyspnea, cyanosis, and loss of consciousness [7]. However, this triad is not universally present [7]. The diagnosis of AFE is a diagnosis of exclusion that relies on clinical presentation, as no reliable gold standard laboratory testing to confirm AFE currently exists. There is growing interest in biomarkers, such as insulin-like growth factor binding protein-1, in aiding in diagnosis [8].

While most often occurring in the setting of labor and delivery [1], AFE have been reported with legal medical and surgical abortions [9, 10], spontaneous abortions [11], and obstetric procedures including amniocentesis [12] and amnioinfusions [13]. If an AFE is suspected while caring for a patient at an outpatient facility, prompt transfer to a hospital should be arranged, with attention to adequately oxygenating and ventilating the patient, and monitoring for signs of hemorrhage and coagulopathy while awaiting transfer. Fluid overload should be avoided as AFE are associated with acute *cor pulmonale* (severely dilated hypokinetic right ventricle), followed by left ventricular failure with risk of cardiogenic pulmonary edema [6].

In this case, coagulopathy was suspected based on ongoing post-procedure vaginal bleeding without evidence of an alternative etiology of hemorrhage, disproportionate oozing from minor trauma to the cervix, lack of clotted blood in the vagina or on perineal-pads in the setting of ongoing vaginal bleeding, and acute hypotension and hypoxia, with clinical concern for an AFE. If there is concern for coagulopathy, the potential for progression to disseminated intravascular coagulopathy should be considered. Traditional laboratory measurements of the coagulation profile were used as the initial evaluation for coagulopathy. Unfortunately, formal laboratory assessments of coagulopathy are time consuming, and in the setting of brisk hemorrhage it is often not appropriate to delay treatment while awaiting results given risk of worsening coagulopathy and blood loss [6, 14]. Moreover, the rapidly evolving coagulation profiles can complicate clinical assessment of laboratory results [4]. For a faster, low-cost bedside assessment of coagulation status, clinicians may use the Lee-White whole-blood clotting test (i.e. “red-top-tube test) [15]. A clotting time longer than 10 min at room temperature is indicative of coagulopathy, while lysis of clot within 1 h suggests fibrinolysis [14]. While useful as an adjuvant to laboratory assessment in quickly determining the presence of coagulopathy, whole-blood clotting tests fail to provide factor-specific guidance. In the setting of clinical evidence of coagulopathy or severe hemorrhage, early initiation of a massive transfusion protocol with 1:1:1 ratios of red blood cells, fresh-frozen plasma, and platelets is recommended without awaiting laboratory assessments [6]. Coordination and close communication with anesthesia colleagues additionally plays a critical role in responding to severe coagulopathy.

While the use of viscoelastic point-of-care coagulation analysis is not widespread in obstetrics, in part due to cost and expertise limitations, there is growing recognition of its usefulness in pregnancy-related coagulopathy management [5, 16, 17]. Our report of coagulopathy in the setting of a suspected AFE following induced abortion adds to several recent case reports highlight the potential utility of viscoelastic point-of-care coagulation analyzers in managing AFE associated coagulopathy at the time of term deliveries [16, 18, 19]. When available, viscoelastic point-of-care coagulation analysis allows for a more efficient and targeted assessment of coagulation compared to traditional laboratory testing or the whole-blood clotting tests [4]. Viscoelastic point-of-care coagulation analysis has different analysis channels that generates a graphical and quantitative representation of whole blood coagulation from clot initiation to lysis, including strength and stability clot assessments. The FIBTEM channel, correlates with plasma fibrinogen levels and clot strength, and indicates whether cryoprecipitate/fibrinogen concentrate therapy should be started [20]. The EXTEM channel tests the extrinsic coagulation pathway: the EXTEM CT correlates with prothrombin time, and therefore informs the clinician as to whether fresh frozen plasma should be given, the EXTEM curve A10 correlates with platelet count and function [20]. The APTEM channel, when compared to the EXTEM channel, gives information as to whether fibrinolysis is ongoing and an antifibrinolytic drug should be administered; if the APTEM and EXTEM graphs look similar, there is not significant fibrinolysis [20].

Viscoelastic point-of-care graphical depictions can be analyzed in real-time, allowing clinicians to gain critical information within minutes of test initiation; A10 results can be visualized 10 min after test initiation as the name implies. In our case, hypofibrinogenemia, which is independently associated with worsened postpartum hemorrhage [5], was diagnosed less than 5 min after ROTEM® initiation, allowing swift administration of fibrinogen repletion before laboratory assessments resulted. Profound hypofibrinogenemia was similarly seen, and correction targeted, in other cases of suspected AFE managed with viscoelastic point-of-care analyzers [16, 18, 19]. Rather than initiating a standard massive-transfusion protocol and awaiting laboratory assessments, when available, viscoelastic point-of-care coagulation analysis use should be considered for tailoring response to pregnancy-related coagulopathy.

## Conclusions

This rare case of suspected AFE and severe coagulopathy following a second trimester surgical abortion highlights the potential advantages of viscoelastic point-of-care coagulation analysis, both in terms of timeliness of intervention and targeting of blood component therapy, in managing coagulopathies resulting from obstetric complications. More research is needed to determine the impact of viscoelastic point-of-care coagulation analysis on patient outcomes in the setting of pregnancy-related coagulopathy.

## Data Availability

The data referred to in this case report was obtained from review of the patient’s medical record and is not publicly available.

## Abbreviations

A10: clot amplitude at 10 min
AFE: Amniotic fluid embolism
aPTT: activated partial thromboplastin time
CBC: complete blood count
CT: clotting time
FFP: fresh frozen plasma
INR: International Normalized Ratio
MCF: mean clot firmness
ROTEM: rotational thromboelastography
TXA: tranexamic acid
